# Use of PET tracers for parathyroid localization: a systematic review and meta-analysis

**DOI:** 10.1007/s00423-016-1425-0

**Published:** 2016-04-16

**Authors:** Wouter P. Kluijfhout, Jesse D. Pasternak, Frederick Thurston Drake, Toni Beninato, Jessica E. Gosnell, Wen T. Shen, Quan-Yang Duh, Isabel E. Allen, Menno R. Vriens, Bart de Keizer, Miguel H. Pampaloni, Insoo Suh

**Affiliations:** 1 Department of Surgery, University of California San Francisco, San Francisco, CA USA; 2 Department of Surgery, University Medical Center Utrecht, Utrecht, Netherlands; 3 Department of Surgery, University Health Network, Toronto, ON Canada; 4 Department of Epidemiology and Biostatistics, University of California San Francisco, San Francisco, CA USA; 5 Department of Radiology and Nuclear Medicine, University Medical Center Utrecht, Utrecht, Netherlands; 6 Department of Nuclear Medicine, University of California San Francisco, San Francisco, CA USA

**Keywords:** Primary hyperparathyroidism, Minimal invasive parathyroidectomy, PET/CT, 11C-Methionine, 18F-Fluorocholine

## Abstract

**Purpose:**

The great spatial and temporal resolution of positron emission tomography might provide the answer for patients with primary hyperparathyroidism (pHPT) and non-localized parathyroid glands. We performed a systematic review of the evidence regarding all investigated tracers.

**Methods:**

A study was considered eligible when the following criteria were met: (1) adults ≥17 years old with non-familial pHPT, (2) evaluation of at least one PET isotope, and (3) post-surgical and pathological diagnosis as the gold standard. Performance was expressed in sensitivity and PPV.

**Results:**

Twenty-four papers were included subdivided by radiopharmaceutical: 14 studies investigated l-[^11^C]Methionine (11C-MET), one [^11^C]2-hydroxy-*N*,*N*,*N*-trimethylethanamium (11C-CH), six 2-deoxy-2-[^18^F]fluoro-d-glucose (18F-FDG), one 6-[^18^F] fluoro-l-DOPA (18F-DOPA), and three *N*-[(^18^F)Fluoromethyl]-2-hydroxy-*N*,*N*-dimethylethanaminium (18F-FCH). The 14 studies investigating MET included a total of 327 patients with 364 lesions. Sensitivity for the detection of a lesion in the correct quadrant had a pooled estimate of 69 % (95 % CI 60–78 %). Heterogeneity was overall high with I^2^ of 51 % (*p* = 0.01) for all 14 studies. Pooled PPV ranged from 91 to 100 % with a pooled estimate of 98 % (95 % CI 96–100 %). Of the other investigated tracers, 18-FCH seems the most promising with high diagnostic performance.

**Conclusions:**

The results of our meta-analysis show that 11C-MET PET has an overall good sensitivity and PPV and may be considered a reliable second-line imaging modality to enable minimally invasive parathyroidectomy. Our literature review suggests that 18F-FCH PET may produce even greater accuracy and should be further investigated using both low-dose CT and MRI for anatomical correlation.

## Introduction

Primary hyperparathyroidism (pHPT) is the third most common endocrine disorder with a prevalence of around 4 per 1000. Incidence rises with age and is twice as common in women. Patients can present with a wide variety of well-described symptoms, ranging from nausea and fatigue to severe osteoporosis and cardiovascular complications [1]. Diagnosis is established biochemically, with the finding of relatively elevated serum calcium (Ca) level and concomitant inappropriately elevated parathyroid hormone (PTH) level. Most commonly, only one of the four glands is abnormal—typically an adenoma—and causes the condition (75–85 %). Less frequently, there are multiple adenomas (15–25 %) and very rarely a carcinoma (1 %) [2]. There is a universal agreement that all patients with symptoms, significant signs of disease (renal or bone manifestations), or young age (<50 years) should undergo surgical exploration [3]. Over the past decade, there has been a shift towards minimally invasive parathyroidectomy (MIP); a focused operation whereby only one parathyroid is removed. Compared to the conventional neck exploration, in which all four glands are investigated intra-operatively, MIP is associated with shorter operating time, lower complication rates, and smaller incision size [4, 5].

Imaging is critical in order to enable successful MIP. Aside from its ability to localize the pathological gland(s), accurate imaging provides valuable anatomical information for the surgeon. ^99^Tc-sestamibi single photon emission computed tomography (SPECT-CT) has become one of the mainstays in first-line parathyroid imaging based on robust evidence demonstrating its ability to predict the location of an adenoma [6]. However, a recent meta-analysis showed that its sensitivity is only 63–84 % (depending on protocol), which leaves a substantial amount of cases with equivocal imaging results [7]. For these cases, several second-line imaging modalities like MRI and CT are used, which have variable performance characteristics. Another modality that has been studied is positron emission tomography (PET). The greater spatial and temporal resolution of PET compared to SPECT imaging allows detection of even the smallest pathological glands, which in theory could improve sensitivity. One of the first reported uses of PET for parathyroid disease used 2-deoxy-2-[^18^F]fluoro-d-glucose (18F-FDG), which revealed the location of a pathological parathyroid gland in a patient with pHPT [8]. Over the years, several other tracers have been investigated as well, with variable results. The performance of PET depends mostly on the ability of the tracer to show specific uptake in the targeted organ. Although PET seems to be a promising new possibility for imaging of pathologic parathyroid glands, not all radiopharmaceuticals are suitable based on multiple factors, including tracer half-life, specificity to parathyroid uptake, and individual hospital characteristics. To our knowledge, there is no pre-existing literature that has reviewed all of the investigated PET tracers in patients with pHPT. Therefore, we performed a systematic review of the evidence available regarding all investigated tracers and also performed a meta-analysis of the data regarding the use of l-(^11^C)Methionine (11C-MET), the most extensively investigated tracer thus far.

## Material and methods

### Literature search

We performed a search of the Embase, PubMed, and Cochrane Library databases published through March 18, 2016, to identify studies investigating the diagnostic value of all PET radiopharmaceuticals for parathyroid localization in patients with biochemical pHPT. We employed an extensive search filter by using all relevant synonyms and Mesh/Emtree terms (Table 1). We completed our search by performing a robust cross-reference check in Web of Science and manually searched references of selected articles, related reviews, and guidelines.

**Table 1:** Search strategy

Strategy component and step no.	Query
PubMed and Cochrane database	
Patient	
1	Hyperparathyroidism OR parathyroid OR HPT OR PHPT
2	MeSH descriptor hyperparathyroidism explode all trees
Intervention	
3	PET OR “Positron Emission Tomography”
4	MeSH descriptor Tomography, Emission-Computed explode all trees
Embase	
Patient	
1	Hyperparathyroidism OR parathyroid OR HPT OR PHPT
2	EMTREE hyperparathyroidism explosion
Intervention	
3	PET OR “Positron Emission Tomography”
4	EMTREE positron emission tomography explosion
Merge	
5	1 OR 2
6	3 OR 4
7	5 AND 6

### Inclusion and exclusion criteria

**Fig. 1:**
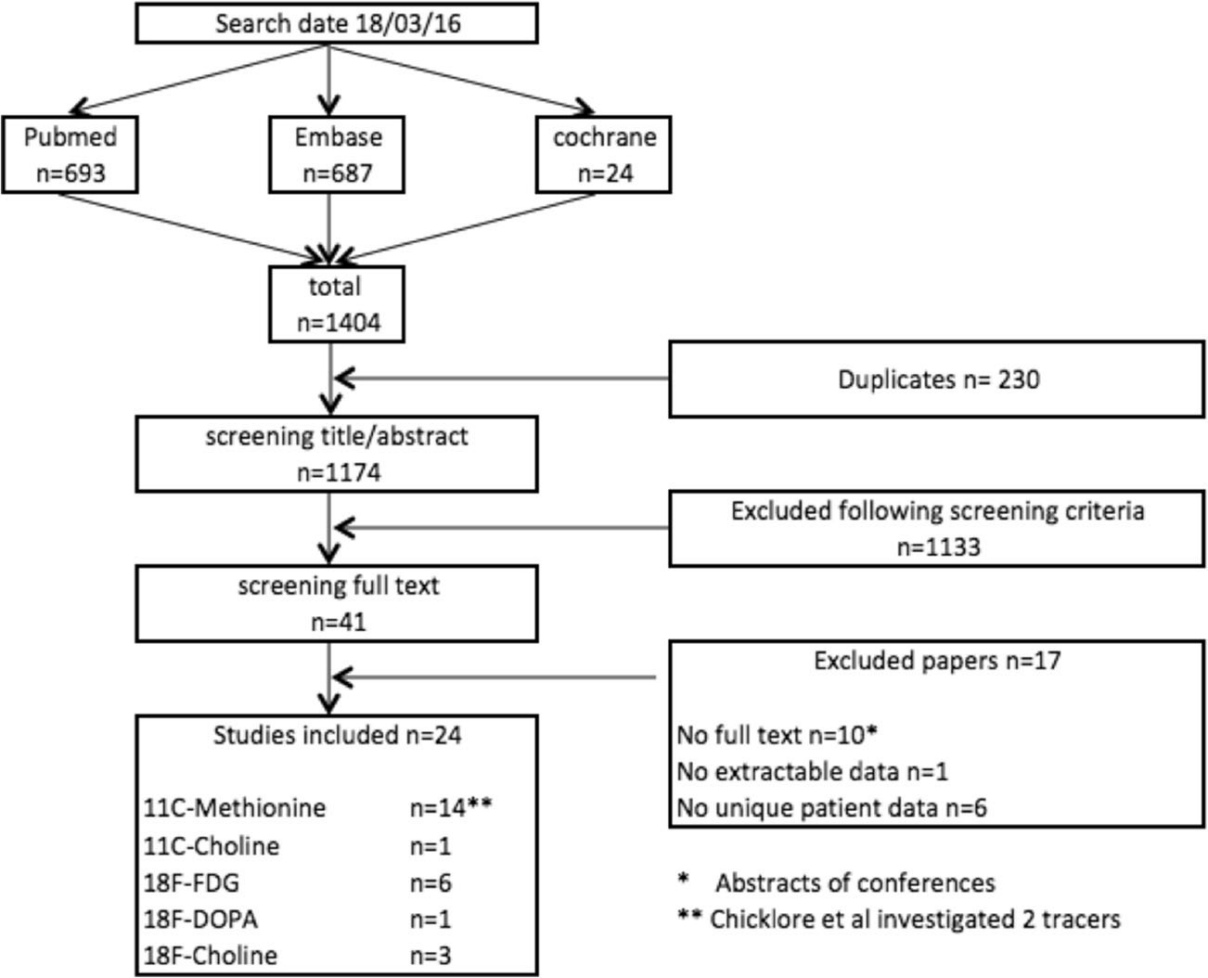
Flowchart of study inclusion

From the publications retrieved via our systematic search, we removed any duplicates (Fig. 1). All remaining unique publications were screened based on title and abstract for eligibility. A study was considered eligible when the following criteria were met: (1) adults ≥17 years old with biochemical non-familial pHPT, (2) evaluation of at least one PET tracer, and (3) post-surgical and pathological diagnosis as the gold standard. If an article presented data for multiple study groups, of which some were eligible for inclusion, the eligible study groups were included if their pertinent data could be extracted.

We excluded studies that differed in design (case reports, systematic reviews), domain (other than pHPT), and language (non-English). All titles and abstracts were screened independently by two authors (W.P.K. and J.D.P.). Any disagreements in selection were discussed, and after consensus, the selected papers were screened for full text review.

Studies were included in the systematic review if they investigated at least five patients, independent of the tracer. Studies were included in the meta-analysis if the following inclusion criteria were met: (1) use of the tracer 11C-MET, (2) extractable and unique data for the calculation of sensitivity and PPV for localization of pathological gland(s) to the correct quadrant (left lower, right lower, left upper, right upper, or ectopic), (3) histological examination of the resected parathyroid gland(s), and (4) a minimum of five included patients.

### Quality assessment

To assess the quality of the included studies, we used the Quality Assessment of Studies of Diagnostic Accuracy Included in Systematic Reviews (QUADAS-)2 tool [9]. This tool is designed to assess the quality of primary diagnostic accuracy studies. It consists of 4 key domains that address (1) patient selection, (2) index test, (3) reference standard and (4) flow of patients through the study and timing of the index tests and reference standard. The domains are assessed for risk of bias and applicability. The two reviewers (W.P.K. and J.P.) used this tool to independently evaluate the methodological quality of the included studies. Disagreements were solved by discussion and thus the final report is based upon consensus of the two reviewers.

### Data extraction

Data were extracted using a standardized spreadsheet. Study design characteristics included the tracer used, injected dose, possible combination with CT, study population, and number of included patients. Since pHPT is a biochemical diagnosis and all patients included were diagnosed with pHPT, there were no true negative scans and therefore specificity and NPV were not calculated. The performance of PET was expressed in sensitivity (total number of glands localized to the correct quadrant / total number of pathological glands found during surgery) and PPV (total number of glands localized to the correct quadrant / total number of glands suspicious for adenomas or hyperplasia). Data regarding MET was calculated using pooled proportion and displayed using forest plots. Sensitivity was defined as the probability that the localization study correctly identified the pathological gland(s). PPV was defined as the probability that a patient with a positive localization study had a pathological gland in that specific location. If data regarding sensitivity or PPV was missing in the selected paper, the author was contacted. Since sensitivity and PPV were calculated using the aforementioned criteria for patients with pHPT, the results may differ from the original papers.

For the studies investigating MET, random-effects models (REM) were used to pool the data [10]. The REM model was fit to account for study heterogeneity. The heterogeneity among studies was tested using the I2 statistic test with a *p* value of less than 0.1 for statistical significance [11]. The I2 statistic is expressed in a percentage scale in which 0 % implies study homogeneity and 100 % indicates that between-study variance is much larger than within study variance. The investigated study population could have influenced the diagnostic performance, thereby causing heterogeneity, and was therefore investigated by subgroup-analysis. The studies were subdivided into 3 groups consisting of patients (1) without specific selection, (2) with negative or discordant conventional imaging, and (3) with a history of previous parathyroid surgery. Funnel plots were used as a visual tool for investigating heterogeneity and publication bias [12]. These plots are scatterplots of the treatment effects estimated from individual studies against a measure of study size. In the absence of publication bias, the funnel plots are symmetrical.

Data analysis was performed using StataSE 13 and conducted by a biostatistician specializing in meta-analysis (I.A.). For this type of study, no formal consent is required.

## Results

### Literature search

The literature search yielded a total of 1174 unique papers, of which 41 papers remained eligible after screening of title and abstract for relevance. After full text review, 24 papers were included. These were subdivided by radiopharmaceutical: 14 studies investigated 11C-MET, one [^11^C]2-hydroxy-*N*,*N*,*N*-trimethylethanamium (11C-CH), six 18F-FDG, one 6-[^18^F] fluoro-l-DOPA (18F-DOPA), and three *N*-[(^18^F)Fluoromethyl]-2-hydroxy-*N*,*N*-dimethylethanaminium (18F-FCH) [13–36]. Excluded were studies that had fewer than five patients, abstracts from conferences without available full text, and redundant studies investigating the same population. The process of study selection is illustrated in Fig. 1. The 14 studies investigating MET underwent meta-analysis, which included a total of 327 patients with 364 lesions.

### Characteristics of the MET studies

All studies were single-center and included between eight and 29 patients, with the exception of the study by Weber et al., which included 102 patients. Four studies had a prospective design, whereas the rest were conducted retrospectively. Most studies also involved patients with secondary (sHPT) or tertiary HPT, or patients that did not proceed to surgery (our gold standard). These patients were excluded from the analysis. Patients with negative or discordant conventional imaging were included in three studies, and two other studies specifically included patients with previous neck surgery. The injected dose of radioactive tracer varied between 370 and 1100 MBq (10–30 mCi) and scanning started between 0 and 40 min after injection with an additional low-dose CT in ten studies. The characteristics are listed in Table 2.

**Table 2:** Characteristics of included studies investigating 11C-Methionine

Study First author	Year	No. of patients		Study population	Dose (MBq)	CT	Time (min)
		Total	pHPT + surgery				
Braeuning	2015	18	12	Neg/disc imaging	600	yes	20
Hayakawa	2015	23	15	Mixed	441–906	yes	15–34
Chicklore	2014	43	15	Mixed	740	yes	10
Martinez	2014	14	14	Mixed	740	yes	10 and 40
Weber	2013	102	102	Mixed	?	yes	20
Chun	2013	16	8	Neg/disc imaging	370–444	yes	30–40
Schalin	2012	21	21	Previous surgery	440	yes	20
Oksuz	2011	8	8	Mixed	700	4/4	15
Weber	2010	33	33	Mixed	?	yes	20
Herrmann	2009	41	9	Mixed	430	no	15–20
Tang	2008	30	22	Mixed	555	yes	20
Beggs	2005	51	29	Neg/disc imaging	619	no	15
Otto	2004	30	14	Mixed	900–1100	no	10
Sundin	1996	34	25	Previous surgery^a^	750	no	direct

*pHPT* primary hyperparathyroidism, *Neg* negative, *Disc* discordant

^a^Previous surgery in 25/34 patients included in the study

### Quality assessment of the MET studies

**Fig. 2:**
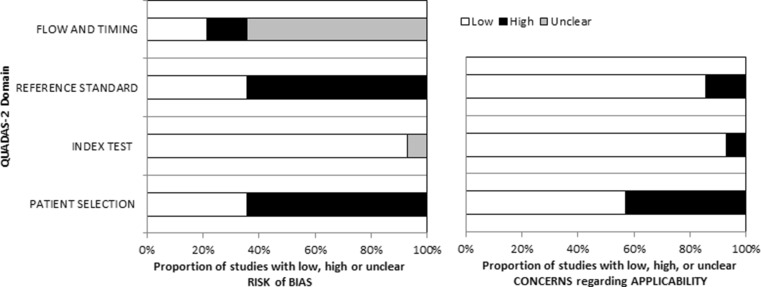
QUADAS 2 tool for risk of bias and applicability

Quality assessment is displayed in Fig. 2 using the QUADAS 2 tool for risk of bias and applicability. Overall, there was a particularly high risk of bias arising from lack of a reference standard and heterogeneity in patient selection. Bias related to the reference standard relates to the fact that a clear definition of cure was lacking in several of the studies. The reference standard varied between no mention of follow-up data to documentation of normocalcemia at 6 months, which is the widely excepted definition of cure. Since estimates of accuracy are based on the assumption that the reference standard is 100 % sensitive and specific, the lack of a clear reference standard might have resulted in misinterpretation of the accuracy of the index test. Substantial risk of bias was present since most studies did not enroll consecutive patients or a random sample of eligible patients. Moreover, as can be seen in Table 2, only a proportion of patients included in the studies underwent surgery. (Because of this, some studies were excluded from the meta-analysis, since they only performed surgery on patients with a positive scan, potentially overestimating its performance.) Concerns regarding applicability were low for the index test, as the scans were usually conducted according to a specific protocol within the studies. However, there was great variability between studies with regard to the PET protocol in terms of tracer dose, use of additional CT, and timing of the scan, which raises concerns as to the applicability of the index test. Patient selection also raises applicability concerns, since the study populations of patients were variable and could not entirely be adjusted for via sub-analysis.

**Fig. 3:**
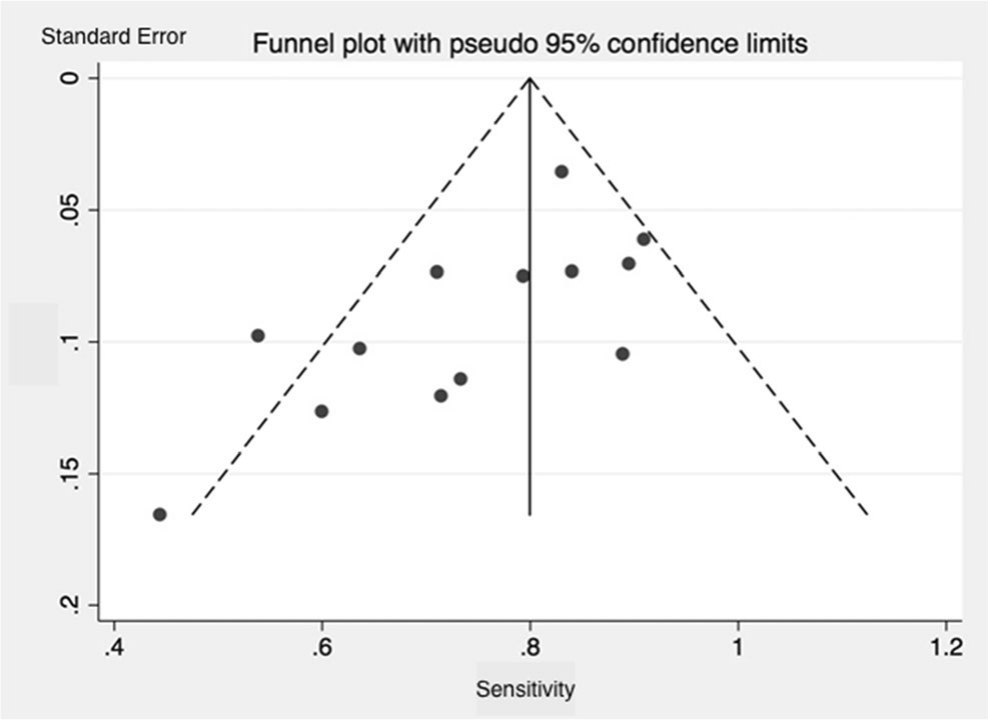
Funnel plot of 11C-Methionine studies included in this meta-analysis. The distribution indicates a publication bias of small and most likely irrelevant studies

Publication bias was investigated using a funnel plot. The distribution indicates a publication bias of small and most likely irrelevant studies (Fig. 3).

### Accuracy and heterogeneity of the MET studies

**Fig. 4:**
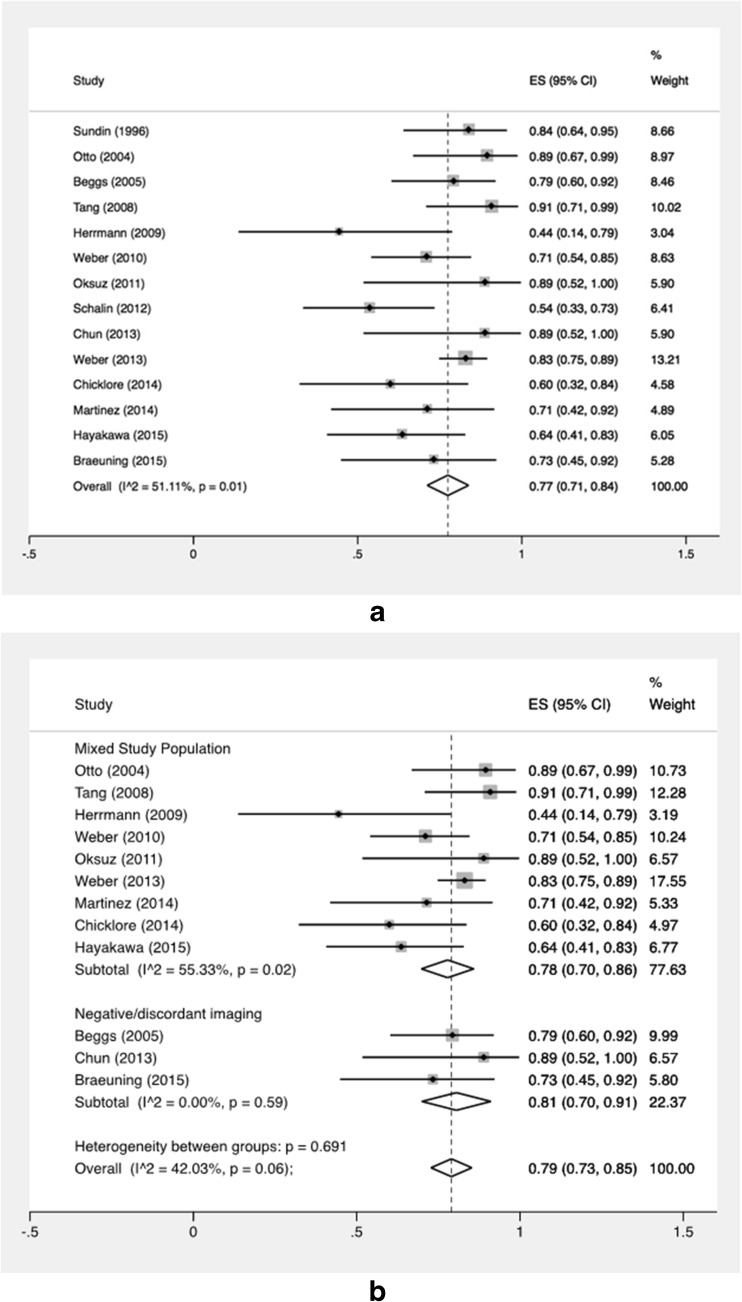
**a** Forest plot of sensitivity of all studies. The pooled result is displayed by the *vertical line. Horizontal lines* indicate the 95 % confidence intervals of each separate study. *Size of the square* is directly linked to the number of patients included in the study. **b** Forest plot of sensitivity of subgroup-analysis depending on domain of included patients. The pooled result is displayed by the *vertical line. Horizontal lines* indicate the 95 % confidence intervals of each separate study. *Size of the square* is directly linked to the number of patients included in the study. *SE* sensitivity, *CI* confidence interval

**Fig. 5:**
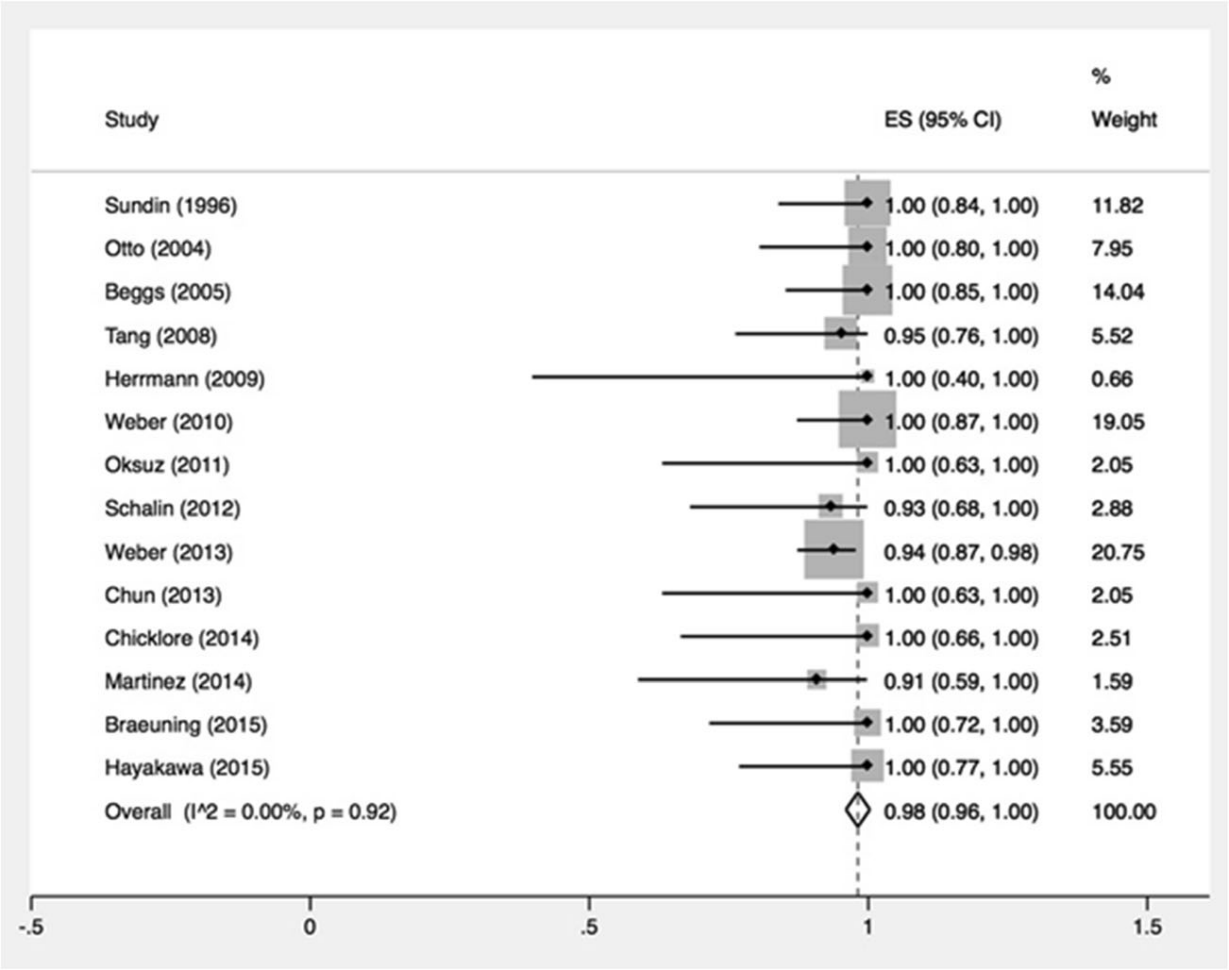
Forest plot of positive predictive value. *SE* sensitivity, *CI* confidence interval

Individual and summary estimates of per-quadrant sensitivity and PPV are shown in Fig. 4a and b. Sensitivity for the detection of a lesion in the correct quadrant ranged from 44 to 91 %, with a pooled estimate of 77 % (95 % CI 71–84 %). Subgroup-analysis based on selection of included patients was performed as well. A total of 9 studies did not specifically select patients (group 1) and showed a random pooled sensitivity of 78 % (95 % CI 70–86 %), which was almost the same as for the three studies that included patients with negative or inconclusive conventional imaging (group 2) and had a pooled sensitivity of 81 % (95 % CI 70–91 %). The two studies that predominantly investigated patients with previous parathyroid surgery had discrepant outcomes with sensitivities of 54 and 84 %. Heterogeneity was overall high with I^2^ of 51 % (*p* = 0.01) for all 14 studies and moderate between the 2 subgroups (I^2^ = 42 %; *p* = 0.06) (Fig. 4a and b). Pooled PPV ranged from 91 to 100 % with a pooled estimate of 98 % (95 % CI 96–100 %) (Fig. 5).

### Other investigated PET tracers

Details about studies investigating other PET tracers are listed in Table 3. From six studies investigating FDG, three were from the same author; however, the domain differed. Two of these included only patients without previous parathyroidectomy—consisting of 17 and 21 patients with pHPT—and excluded patients with hyperplasia from the results [33, 36]. These studies found sensitivities of 94 and 86 % and PPVs of 85 and 86 %, respectively. The third included 20 patients with a history of neck surgery and found a much lower sensitivity and PPV, both 62 % [32]. The three other studies found even lower sensitivities, varying between 0 and 27 %, although PPVs were consistently high. Notably, none of the 8 scans performed by Sisson et al. showed a focus that could be attributed to a parathyroid adenoma [35].

**Table 3:** Characteristics of studies investigating PET tracers other than 11C-Methionine

Study First author	Tracer	Year	No. of patients		Dose (MBq)	CT	Time (min)	Sens (%)	PPV (%)
			Total	pHPT + surgery					
Kluijfhout	18F-FCH	2015	5	5	2 MBq/kg	yes	direct	80	100
Michaud	18F-FCH	2015	17	11	3 MBq/kg	yes	direct	94	91
Lezaic	18F-FCH	2015	43	43	100	yes	5,60,120	95	97
Orevi	11C-CH	2014	40	?	370	yes	direct	?	?
Lange-Nolde	18F-DOPA	2006	8	8	185–300	no	90	–	–
Chicklore	18F-FDG	2014	43	15	400	yes	90	27	100
Neumann	18F-FDG	1997	20	20	185–370	no	45	62	62
Neumann	18F-FDG	1996	21	21	185–370	no	45	86	86
Melon	18F-FDG	1995	7	7	370	no	50	22	100
Sisson	18F-FDG	1994	8	8	370	no	?	0	–
Neumann	18F-FDG	1994	17	17	185–370	no	45	94	85

*pHPT* primary hyperparathyroidism, *Sens* sensitivity, *PPV* positive predictive value

Five recent studies published results regarding 18F-FCH; however, Michaud et al. and Lezaic et al. published two articles using the same population [28, 29, 37, 38]. The most recent study of Michaud et al. included 17 patients without prior neck surgery, divided in 11 patients with pHPT, five with sHPT, and one lithium-associated [28]. All patients had 18F-FCH PET-CT because of negative/discordant ultrasound and ^123^I/^99m^Tc-sestamibi subtraction scintigraphy. Sensitivity per lesion was up to 94 %. The most recent study of Lezaic et al. included 43 patients with pHPT and no previous neck surgery [29]. All patients underwent ultrasound, ^99m^Tc-sestamibi/pertechnetaat subtraction scintigraphy, SPECT-CT, and 18F-FCH PET-CT. Sensitivity per lesion of PET-CT was up to 95 %. Kluijfhout et al. conducted 5 scans in patients with negative conventional imaging and found a sensitivity of 80 % with a PPV of 100 % [27].

Lange-Nolde et al. investigated the performance of 18F-DOPA [31]. They included eight patients with pHPT. All had histologically proven adenomas; however, none of the scans showed any detectable uptake.

Orevi et al. published preliminary results of 11C-CH PET-CT [30]. They included 40 patients with HPT, of which 20 were diagnosed with pHPT and the latter predominantly with sHPT. In 24 of 27 patients that underwent surgery so far, there was concordance between PET result and surgical findings. There was insufficient data to calculate specific performance for patients with pHPT; however, the scan was clearly positive in 37 out of 40 patients.

## Discussion

We performed a meta-analysis to investigate the performance of 11C-MET PET to localize pathological parathyroid gland(s) in patients with pHPT. Our results show a pooled sensitivity and PPV for the detection of a pathological parathyroid in the correct quadrant of 77 and 98 %, respectively. In subgroup-analysis, we found no difference in sensitivity in patients without specific selection versus patients with negative/discordant conventional imaging (78 versus 81 %, respectively). Among the other investigated tracers in our systematic review, 18F-FCH seems the most promising with sensitivity ranging from 80–100 % and PPV 89–100 % in three studies with a total of 37 patients.

Our pooled sensitivity of 77 % is slightly lower than the pooled sensitivity of 81 % found by Caldarella et al. who published a meta-analysis regarding 11C-MET PET in 2012 [39]. Since then, new evidence has been published and there are several major methodological differences between that study and the current meta-analysis. First, Caldarella et al. did not solely include patients with pHPT but also with sHPT. Second, their calculations are based on a per-patient level: as most surgeons favor MIP to bilateral neck exploration, a per-lesion sensitivity for localization to the correct quadrant may be more clinically relevant. Identification of the right quadrant per lesion is more precise, which might explain our slightly lower pooled sensitivity. Lastly, PPV is not calculated; instead, the authors presented a detection rate, defined as percentage of positive scans, without correlating this to the surgical outcome. In an attempt to examine a more homogenous group, we included only patients with pHPT. The domain of the included patients did however still vary greatly, which is why subgroup-analysis was performed to examine whether patient domain influenced the accuracy of 11C-MET PET.

In subgroup-analysis, performance of 11C-MET PET in a mixed patient cohort (group 1; pooled sensitivity 78 %) was comparable to conventional ^99m^TC-sestamibi SPECT (sensitivity 79 %), but substantially lower compared to sestamibi SPECT/CT (sensitivity of 88 %) [40, 41]. Remarkably, the performance of 11C-MET PET did not decline significantly when only patients with negative or inconclusive imaging were included (group 2, pooled sensitivity 81 %). This suggests that 11C-MET PET is relatively most useful in a substantial amount of cases with negative/discordant conventional imaging and could therefore be considered as a suitable second-line imaging modality.

The ability of 11C-MET PET to detect hyperplastic glands was lower compared to patients with adenomas. In the largest study included in our meta-analysis, overall sensitivity was 83 %, versus 33 % for hyperplastic glands [17]. Hyperplastic glands are often smaller in size and weigh less than adenomas, both factors that have previously been associated with decreased sensitivity [14]. This might also be a contributing reason as to why performance is lower in patients with persistent pHPT after previous parathyroid surgery. Schalin et al. included 21 patients with pHPT and persistent disease and found a sensitivity of 44 %, substantially lower than the pooled sensitivity of 77 % found in this study [19]. Weber et al. also performed a subgroup-analysis on patients with previous neck surgery, including patients after thyroid surgery. Remarkably the sensitivity in this group was higher compared to the total group (94 versus 83 %). Thyroid tissue reduces the lesion-to-background ratio due to physiological thyroidal uptake of 11C-MET and thereby complicates reading of parathyroid scans [22]. Pathological parathyroids that are in a juxtathyroidal location can therefore cause false negative outcomes [26].

Detection of accumulated 11C-MET can be done at different times after injection, and protocols varied in this study from scanning immediately after injection to scanning 40 min post-injection. Two studies had protocols in which patients were scanned twice at 10 and 40 min to compare the effect of timing on diagnostic efficacy [16, 25]. They found the best parathyroid to background contrast and the best delineation of hyper-functioning parathyroid after 10 min, although the best parathyroid to thyroid contrast was seen after 40 min. Martinez et al. included the late scan when the 10-min scan was negative, which resulted in the detection of one additional positive parathyroid gland in their study. Scans later than 40 min resulted in insufficient count statistics owing to the short half-life of 11C-MET (20 min). The short half-life of 11C-MET and the complicating labeling procedure is also one of the biggest limitations of MET. It requires the center to have a cyclotron for production of the tracer since transportation from another facility would take too much time.

Recent publications often used a combination of PET and CT. Contrast enhancement CT can be useful to differentiate with lymph nodes and might therefore prevent false positive outcomes [42]. However, there is also increasing attention to the possible risks of extra radiation involved with CT [43]. Several studies also investigated other factors that may affect the accuracy, such as goiter, thyroiditis, and parathyroid hormone level; however, a separate analysis could not be performed due to insufficient available data. Nevertheless, in general PET appears to work well even in these settings [17, 26].

The second best investigated tracer is 18F-FDG, which has the advantage of a significant longer half-time of 110 min, enabling off-site production. Also, it is by far the most commonly used PET radiopharmaceutical and has wide availability. These benefits notwithstanding performance varied greatly between the six studies that have investigated its use with a sensitivity of 0–94 % and PPV of 62–100 %. Due to these highly discrepant findings, FDG appears to be a less useful tracer for the detection of pathological parathyroids. 18F-DOPA, in a limited trial, performed even more poorly. Using a prospective study design, Lange-Nolde et al. scanned eight patients with pHPT, each of whom had adenomas detected in the operating room; however, none of the 18F-DOPA PET scans were positive [31]. More promising seems to be the use of 11C-CH. A recent prospective study by Orevi et al. included 40 patients with HPT, and there were 37 choline positive scans [30]. Although their results are preliminary and only some of the patients have undergone surgery, these data suggest that 11C-CH may be an important tracer in radiographic localization of parathyroid adenomas. The main disadvantage (similar to 11C-MET) is the short half-life of 20 min, which obligates on-site production. This problem has been overcome by the most promising PET tracer thus far, 18F-FCH, which has a half-life of 110 min. This tracer is frequently used in the diagnostic work-up and follow-up of patients with prostate cancer and has, therefore, wide availability. So far, three studies have published unique data with excellent preliminary results, even in patients with negative SPECT-CT [27–29]. The advantage of 18F-FCH PET-CT over SPECT-CT is the higher spatial resolution, lower radiation burden (around 6 versus 8 mSv), and the substantially shorter scanning time with a single acquisition of around 10 min, depending on protocol [44].

Besides PET, several other modalities have been studied for use as a second-line imaging as well. Computed tomography is available in virtually every medical center and has been shown to accurately detect parathyroid adenomas, even in the case of negative conventional imaging [45, 46]. There is increasing attention to 4D-CT, which can help clinicians differentiate adenomas from other structures in the neck [47]. Performance has shown to be good, with a sensitivity and PPV of around 80 and 90 %, respectively, depending on the investigated population [48, 49]. The major drawback of this technique, which uses multiple phases, is radiation exposure that can be as high as 26 mSv from a single scan [50]. MRI can also be used to localize parathyroid glands. Evidence remains scarce, but one of the largest studies so far showed a sensitivity of 82 % with a PPV of 89 % and a very recent publication also showed promising results [51, 52]. Considering the unique benefits of 18F-FCH PET and MRI, a potentially superior modality would be using the 18F-FCH tracer and a combined PET MRI scanner. Although no studies have been published yet, preliminary results from a pilot-study show excellent results in patients with negative or discordant conventional imaging [Unpublished results Kluijfhout et al.].

Our meta-analysis has several limitations. Like any meta-analysis, its quality is limited by the number and quality of constituent studies. Each individual study included only a small number of patients. As shown by the QUADAS 2 tool, there was also a substantial risk of bias arising from variability in patient selection and reference standard. Moreover, there is concern regarding applicability as various imaging protocols were used. The results of this meta-analysis should therefore be interpreted with caution, and more research, especially in the form of prospective studies, is needed to confirm our observations.

## Conclusion

To our knowledge, this is the first systematic review investigating all PET tracers that have been used for the detection of pathological parathyroid glands. The results of our meta-analysis show that 11C-MET PET has an overall good sensitivity and PPV and may be considered a reliable second-line imaging modality to enable minimally invasive parathyroidectomy. Our literature review suggests that 18F-FCH PET may produce even greater accuracy and should be further investigated using both low-dose CT and MRI for anatomical correlation.
